# Exploring Patient-Centered Perspectives on Suicidal Ideation: A Mixed-Methods Investigation in Gastrointestinal Cancer Care

**DOI:** 10.3390/cancers17152460

**Published:** 2025-07-25

**Authors:** Avishek Choudhury, Yeganeh Shahsavar, Imtiaz Ahmed, M. Abdullah Al-Mamun, Safa Elkefi

**Affiliations:** 1 Industrial and Management Systems Engineering, Benjamin M. Statler College of Engineering and Mineral Resources, West Virginia University, Morgantown, WV 26506, USA; 2 Department of Pharmaceutical Systems and Policy, School of Pharmacy, West Virginia University, Morgantown, WV 26505, USA; 3 Columbia University School of Nursing, Columbia University Irving Medical Center, New York, NY 10032, USA; 4 School of Systems Science and Industrial Engineering, Watson College of Engineering Binghamton, Binghamton University, Vestal, NY 13902, USA

**Keywords:** gastrointestinal cancer, patient perspectives, qualitative study, cancer care, support, care quality, information, financial burden

## Abstract

Background: Gastrointestinal (GI) cancer patients face a four-fold higher suicide risk than the general US population. This study explores psychosocial aspects of GI cancer patient experiences, assessing suicidal ideation and behavior, mental distress during treatment phases, and psychosocial factors on mental health. Methods: A two-phase mixed-methods approach involved a web-based survey and follow-up interviews. Quantitative data analysis validated mental health and suicidal ideation constructs, and correlation analyses were performed. The patient journey was charted from diagnosis to treatment. Results: Two hundred and two individuals participated, with 76 from the rural Appalachian region and 78 undergoing treatments. Quantitative analysis showed a higher prevalence of passive suicidal ideation than active planning. The post-treatment recovery period was the most emotionally challenging. Qualitative data emphasized emotional support and vulnerability to isolation. Care quality concerns included individualized treatment plans and better communication. Patients also needed clear, comprehensive information about treatment and side effects. The in-depth interview with four GI cancer patients revealed a healthcare system prioritizing expedient treatment over comprehensive care, lacking formal psychological support. AI emerged as a promising avenue for enhancing patient understanding and treatment options. Conclusions: Our research advocates for a patient-centric model of care, enhanced by technology and empathetic communication.

## Simple Summary

Many patients diagnosed with gastrointestinal (GI) cancer experience emotional struggles, including feelings of depression and suicidal thoughts, yet these psychological needs often remain overlooked. This study was designed to better understand the mental health challenges faced by GI cancer patients—especially after treatment ends—by listening directly to their experiences through surveys and interviews. The findings reveal that the post-treatment phase can be more emotionally distressing than the diagnosis itself, with feelings of isolation, family burden, and lack of support contributing to poor mental health. The study also shows that access to emotional support, clear communication from doctors, and trusted health information—possibly delivered through AI—can improve patients’ psychological well-being. By shedding light on these needs, this research encourages more compassionate, patient-centered cancer care that addresses both physical and mental health.

## 1. Introduction

Suicide constitutes a pressing global public health issue, exerting a substantial impact on mortality rates across the world [1]. The World Health Organization (WHO) reports an estimated 703,000 annual suicide-related deaths worldwide. Moreover, incidents associated with suicide, such as contemplation of suicide, suicide attempts, devising suicide plans, and impulsive thoughts of self-harm, can inflict adverse psychological consequences on both individuals and those in their immediate social circles [2].

According to the WHO’s 2019 assessments, cancer emerges as one of the leading cause of premature death before the age of 70 across 112 nations [3]. One of the most disturbing factors linked with cancer is the significantly increased risk of suicide among patients. This issue has attracted considerable attention from the medical community and society [4,5,6,7,8,9,10,11]. Studies reveal that in gastrointestinal (GI) cancer, a group of cancers that affects the gastrointestinal tract and associated organs (digestive system), patients face a four-fold higher risk of suicide than the general US population, with suicide ideation and completion being most prevalent within the first six to twelve months post diagnosis [7,12,13]. This disturbing trend has prompted several health organizations to take notice. The American College of Surgeons Committee on Cancer, the American Society of Clinical Oncology, and the National Comprehensive Cancer Network have all stressed the importance of addressing cancer patients’ psychological and emotional needs [14,15]. While efforts to reduce suicide risk in cancer patients have been crucial, the issue remains urgent.

The psychological impact of GI cancer, including depressive disorders and suicidal ideation, has been well documented, with studies highlighting the strong relationship between psychological characteristics and internet use, family burden, household occupancy, age, and emotional support [16]. Furthermore, coping style, insomnia, and psychological distress have been identified as important factors affecting the well-being of individuals with gastrointestinal cancer, emphasizing the need for a holistic approach to address the psychological aspects of this patient population [17]. The prevalence of suicidal ideation in cancer patients, including those with gastrointestinal cancers, has been a subject of research, with studies reporting a higher risk of suicidal ideation in specific cancer subtypes, such as ovarian cancer [18]. The distress experienced by cancer patients, as measured by tools like the Distress Thermometer, has been found to have predictive value for assessing the risk of suicide in this population, highlighting the need for proactive psychological support and intervention [19].

In cancer care, acknowledging and incorporating the voices, perspectives, and needs of GI cancer patients is increasingly recognized as a critical aspect of comprehensive healthcare. This perspective emphasizes the necessity of understanding and addressing the unique challenges faced by individuals with GI cancer, aiming to enhance both their treatment outcomes and overall quality of life.

As healthcare providers and researchers strive to optimize care, the importance of patient-centered approaches becomes self-evident. There is a lack of studies exploring the mental health struggles experienced by GI cancer patients. In this study, we explore the needs and stories of GI cancer patients, emphasizing the potential impact of personalized, holistic care on suicidal ideation and mental distress in affected individuals. Our investigation seeks to explore patient perspectives, offering insights that could reshape the landscape of cancer care. By doing so, we aim to foster a more compassionate, comprehensive, and practical approach to addressing the psychological challenges often accompanying a GI cancer diagnosis.

## 2. Materials and Methods

The study received ethical approval from the West Virginia University Institutional Review Board under protocol number 2212691613. The study qualified for the WVU Flexibility Review Model, as it involves minimal risk and adheres to the Belmont Report’s ethical principles. Approval was granted on 7 February 2023.

### 2.1. Study Design and Data Collection

The data for this study were collected in two sequential phases (A and B) through a validated web-based survey and semi-structured interviews, respectively. Any individual in the US with GI cancer (undergoing or completed treatment) was eligible to participate in this study.

**Figure 1:**
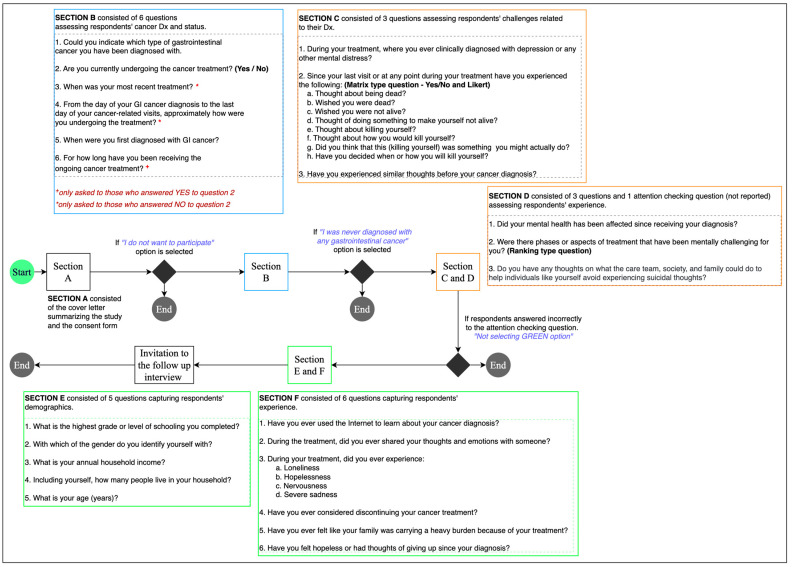
Survey design and flow.

In phase 1, a web-based survey (see Figure 1) was distributed to cancer patients through social media and open cancer forums via an audience paneling service, Centiment. Centiment reaches a broader and more representative audience via its network and social media. They also use fingerprinting technology that combines IP address, device type, screen size, and cookies to ensure that unique panelists enter the survey.

The survey contained validated questions adapted from the Columbia Suicide Severity Rating Scale (C-SSRS) [20] and the Patient Health Questionnaire (PHQ-4) [21,22]. PHQ questions were reverse coded, where higher score indicates better mental health. Other variables, such as family burden, internet information utilization, and emotional support, were measured using a five-point Likert scale. The survey was accompanied by a detailed cover letter, an electronic consent form, and an invitation for an online follow-up interview. After reviewing the cover letter and consent form, individuals with gastrointestinal issues who agreed to participate were directed to the survey. Participant eligibility was determined through self-reported data. A control question was included to maintain response integrity: “To confirm attentive reading and thoughtful responses, please select Green as your answer.” Responses failing this criterion were excluded from the analysis.

Participants were debriefed and invited for a follow-up interview after completing the survey. The principal investigator’s (PI’s) contact information was provided, encouraging participants to reach out if interested in the interview phase. Four individuals contacted the PI and agreed to participate in the follow-up interview (i.e., Phase 2).

**Figure 2:**
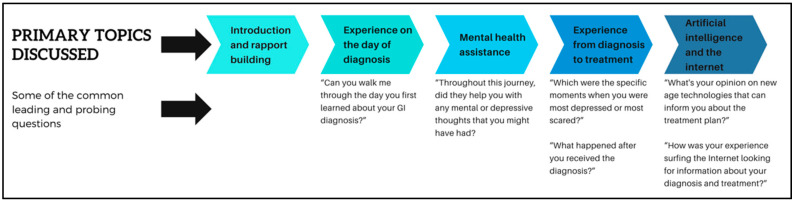
The overall interview processes.

Phase 2 started soon after the completion of Phase 1. The PI interviewed the participants over Zoom for approximately 45 min per participant. Both the parties had their web camera switched on. The interview sessions were recorded and manually transcribed for analysis. After each interview, the participants were emailed a USD 50 Amazon gift card as an honorarium. No personal identifiers were collected. All names and identifiers in the transcription were anonymized before analysis. Figure 2 shows the overall interview process.

### 2.2. Quantitative Analysis

The descriptive statistics of all the variables were calculated in R (JASP Version 0.19.3). To ensure validity and reliability of the survey measures, the questions adapted from PHQ-4 and C-SSRS were converted into latent constructs, namely mental health (Factor 1) and suicidal ideation (Factor 2), respectively. The constructs were validated using multigroup confirmatory factor analysis across both groups (participants from rural Appalachia and others). Model fit was assessed by the comparative fit index (CFI) [23], Tucker Lewis index (TLI) [24], and Bentler Bonnet Normed fit index (NFI) [23]. Additional measures such as the parsimony normed fit index (PNFI) [25], Bollen’s relative fit index (RFI) [26], Bollen’s incremental fit index (IFI) [26], and relative non-centrality index (RNI) [27] were also observed. Reliability and validity were assessed using Cronbach’s alpha requiring to be greater than 0.70; (b) outer loadings greater than 0.50 [28] Fornell–Larcker criterion [29] and the Heterotrait–Monotrait (HTMT) ratio required to be less than 0.85 [30]. The reliability and validity outcomes are reported in Supplementary Materials.

Upon validating the latent constructs, the scores of each construct were extracted, and the dataset was scaled. Given the non-normal distribution of the data (based on the Shapiro–Wilk test), we used Bayesian Pearson’s Rho correlation analysis as it is more robust to non-normality than the classical Pearson correlation.

### 2.3. Qualitative Thematic Analysis

An inductive thematic analysis was employed to analyze the open-ended survey responses and the in-depth interview script. Three coders (two with experience in qualitative data analysis and one with expertise in the subject matter) reviewed each response. Upon discussing with each other, they assigned tentative labels (or codes) in Microsoft Excel [31], a process called open coding [32]. The coders then reviewed the responses and open codes to identify relationships and similarities between codes, a process known as axial coding [33]. The codes were merged into broader themes representing the core concepts, called selective coding [34]. All coders engaged in iterative discussions to refine and finalize a set of consensus codes, ensuring that the identified themes accurately captured the essence of the participants’ experiences and perspectives.

### 2.4. Case Study Analysis

Additionally, the in-depth interview transcripts were analyzed to chart the patient’s experience, beginning with the day they received a cancer diagnosis and continuing through the entire treatment and recovery process. First, three coders iteratively read the transcripts to understand participants’ perceptions of their journey better. Then, with discussion and consensus, the team of coders matched the participants’ responses with the phase in their cancer journey. The diagnosis phase section described the patients’ first encounters with the healthcare system, detailing the process of obtaining a diagnosis and the initial comprehension of their medical condition. The treatment phase discusses the various treatments patients undergo, such as surgery, chemotherapy, and radiation, and addresses the challenges encountered, including treatment resistance and side effects. The emotions phase examines the psychological effects of cancer diagnosis and treatment, exploring the emotional responses and the support received from healthcare professionals, family, and friends. Lastly, the communication and artificial intelligence phase considers the role of communication in shaping the treatment experience and investigates how artificial intelligence could enhance patient care and communication.

## 3. Results

### 3.1. Participant Characteristics

Two hundred and two individuals participated in the study. Of all the participants, 76 respondents were from the rural Appalachian region, and 78 were undergoing treatment during the study. About 73 respondents were not clinically diagnosed with mental distress during treatment. A majority of the participants reported that they had never experienced mental distress before their cancer diagnosis, with 107 individuals selecting “never.” A smaller number, 52 respondents, indicated that they had rarely experienced such thoughts or emotions. Meanwhile, 32 participants noted that they sometimes had similar feelings in the past, and a smaller contingent—8 respondents—reported feeling this way most of the time. Only 3 participants stated that they always experienced such thoughts or emotions prior to their diagnosis.

**Figure 3:**
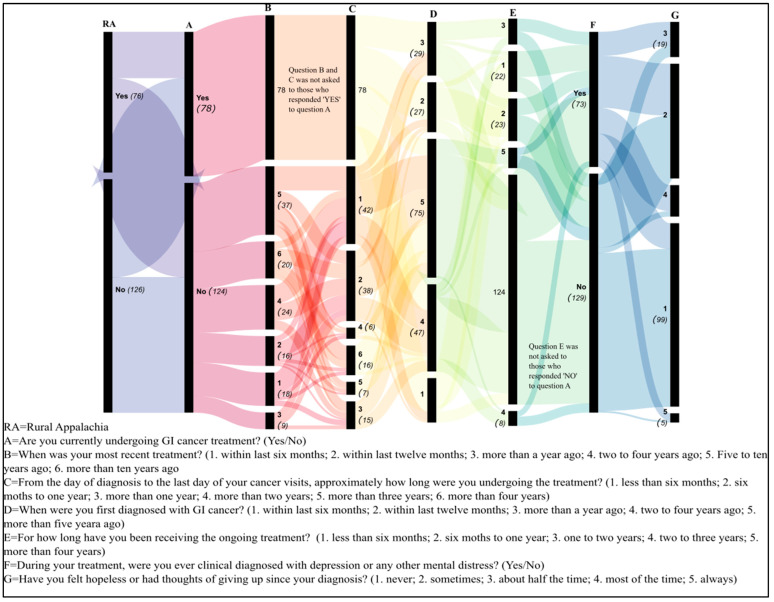
Sankey diagram illustrates the flow and distribution of participant responses through a set of sequential surveys with conditional branching. Each vertical band represents a different question from the survey, with the width of the bands and connecting ribbons proportional to the number of responses. The leftmost band (RA) bifurcates respondents into two categories, ‘from rural Appalachia (‘Yes’ 76) or not (‘No’ 126), with subsequent questions contingent upon prior answers. The thickness of each flow represents the number of respondents transitioning from one question to the next.

The demographic trends indicative of the diversity within the patient population emerged. Educational level varied across the cohort, with a substantial number achieving some college-level education. Notably, 54 individuals reported some college education without obtaining a degree, while 50 completed a 4-year degree. Those with professional degrees numbered 25, and 6 individuals had attained a doctorate level of education. Income levels demonstrated an economic spread across the cohort, with a significant number of participants (44) reporting annual household incomes between USD 20,000 and <USD 35,000, followed closely by those earning USD 50,000 to <USD 80,000 (42 individuals). Fewer participants reported incomes at the extremes, with 23 individuals earning less than USD 20,000 annually and 7 earning more than USD 200,000, underscoring the economic diversity within the study group. Age demographics highlighted a concentration of older adults, with 72 participants older than 65, the largest group in the cohort. The remaining age groups were more evenly distributed, with the 46 to 55 year-old and 56 to 65 year-old categories being the next most populous, with 54 and 41 individuals, respectively. Notably, the younger age groups (18 to 24 and 25 to 35) were represented by only one individual each, indicating a predominance of middle-aged to older adults in the study. Figure 3 illustrates the response flow among a set of survey questions.

### 3.2. Quantitative Findings

**Figure 4:**
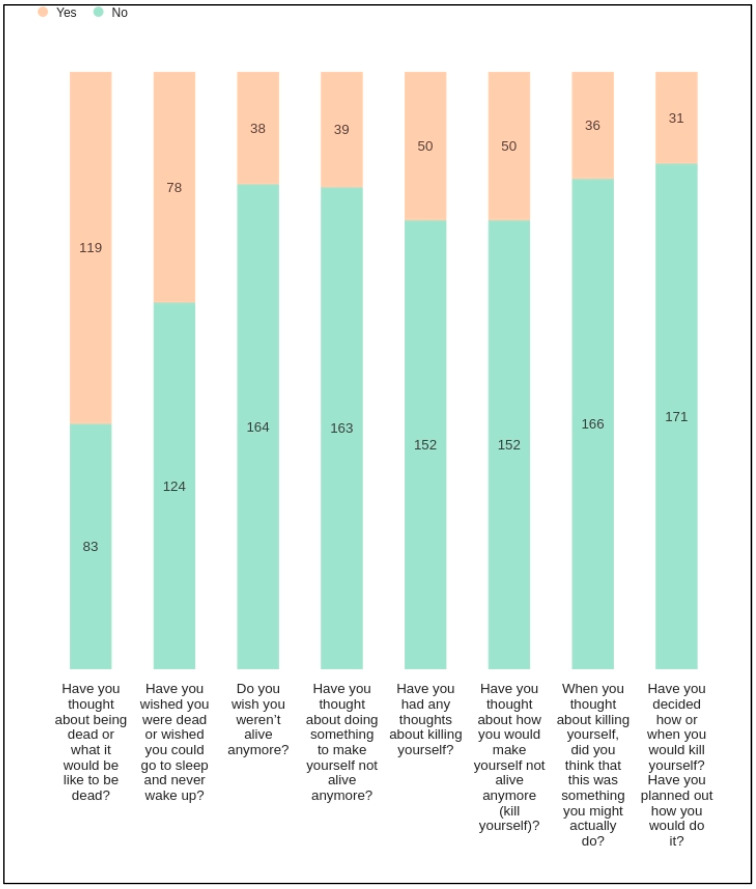
Distribution of responses to questions on suicidality. The figure presents a stacked bar chart depicting participant responses to a series of questions related to suicidality. Each bar represents the number of ‘Yes’ and ‘No’ responses to individual questions, with the total height corresponding to the total number of respondents. The questions progress from less severe ideation (e.g., thinking about being dead) to more severe considerations (e.g., planning how to kill oneself). The colors differentiate between ‘Yes’ responses (peach) and ‘No’ (teal), with the respective counts provided within the bars.

Figure 4 demonstrates the prevalence of suicidal ideation and behavior among study participants (GI cancer patients). Initial questions regarding passive suicidal ideation, such as the desire to be dead or not wake up, elicited a higher number of affirmative responses compared to more active forms of ideation, such as having a suicide plan. The data reveal an intriguing decline in affirmative responses as the questions probe more concrete steps toward suicidal action. For example, while 78 individuals contemplated death, only 31 had progressed to formulating a specific plan. This decrement underscores the potential for intervention strategies to disrupt the progression from ideation to action.

**Figure 5:**
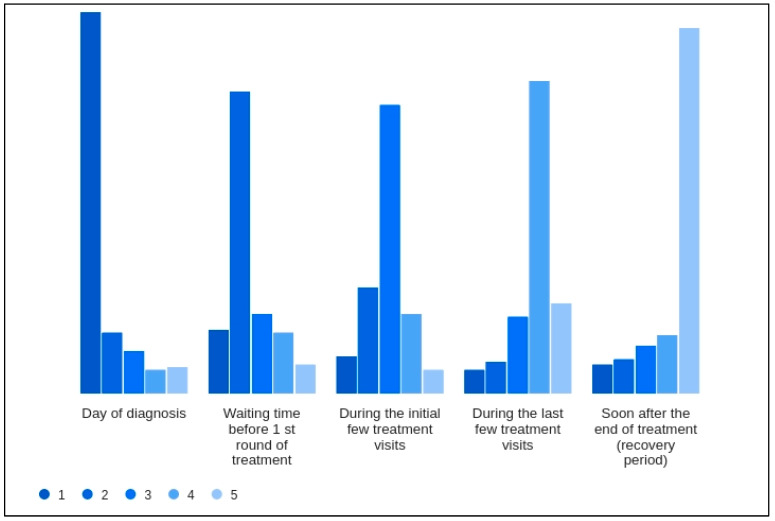
Patient-reported mental distress during different phases of cancer treatment. The histogram illustrates the distribution of mental distress rankings assigned by 202 cancer patients to five distinct phases of their cancer treatment journey. Each phase is evaluated on a scale from 1 (least distressing) to 5 (most distressing). The results show that the ‘Day of diagnosis’ is most frequently ranked as least distressing, whereas the ‘Soon after the end of treatment (recovery period)’ is predominantly ranked as the most distressing phase. This counterintuitive finding highlights patients’ complex emotional landscape and underscores the need for supportive care throughout the treatment continuum, especially post-treatment.

Figure 5 presents a histogram, with each bar representing the number of cancer patients who ranked particular phases of their treatment journey according to the level of mental distress experienced, from 1 (least distressing) to 5 (most distressing). It indicates that patients may find the recovery period after the end of treatment to be the most challenging in terms of mental and emotional health, which could inform healthcare providers about when to offer increased mental health support. Conversely, the day of diagnosis, which might be assumed to be highly distressing, appears to be less so for the respondents in this survey. This could reflect various factors, including relief at having a precise diagnosis or effective initial counseling and support.

Table 1 presents descriptive statistics for study variables. The analysis of the mean scores for suicidal ideation (SI 1–8) items reveals significant insights into the prevalence (as shown in Figure 4) and intensity (mean reported in Table 1) of these thoughts among the surveyed individuals. For instance, when both the mean SI scores (Table 1) and the number of ‘Yes’ responses in the corresponding Figure 4 are high, it suggests that a specific aspect of suicidal ideation is commonly experienced among respondents. This convergence indicates a shared tendency toward such thoughts, pointing to a generalized concern that may warrant public health attention. Conversely, lower mean SI scores (Table 1), when juxtaposed with a substantial number of ‘Yes’ responses (Figure 4), suggest a more polarized experience within the population. This dichotomy implies that while the average level of suicidal ideation may be moderate, there exists a subset of individuals experiencing intense levels of such ideation, thereby revealing a hidden severity not immediately apparent in the mean score alone.

Furthermore, the standard deviations (Std. Dev.) and variances suggest moderate response variability. Notably, the 95% confidence intervals for mean scores of SI items remained narrow, reflecting a high level of precision in the measurement of central tendency. However, a negative skewness in the latter items (SI 3–8) suggests a tendency towards lower frequencies of more severe suicidal ideation, with fewer respondents contemplating concrete plans or actions.

The mental health experiences depicted by MH 1–4 (Table 1) show a similar pattern of narrow confidence intervals and low skewness, with mean scores indicating less distress than the SI items. However, the negative skewness in three out of four MH items suggests that a significant number of individuals are experiencing less favorable mental health states, which the averaging effect of the mean could mask.

The utilization of internet information (Internet Info Util) and seeking emotional support have mean scores around the mid-range of the scale, suggesting a moderate engagement with these resources among respondents. The skewness in the family burden item is notably positive, indicating that many respondents perceive their family as facing a substantial burden due to their treatment.

**Figure 6:**
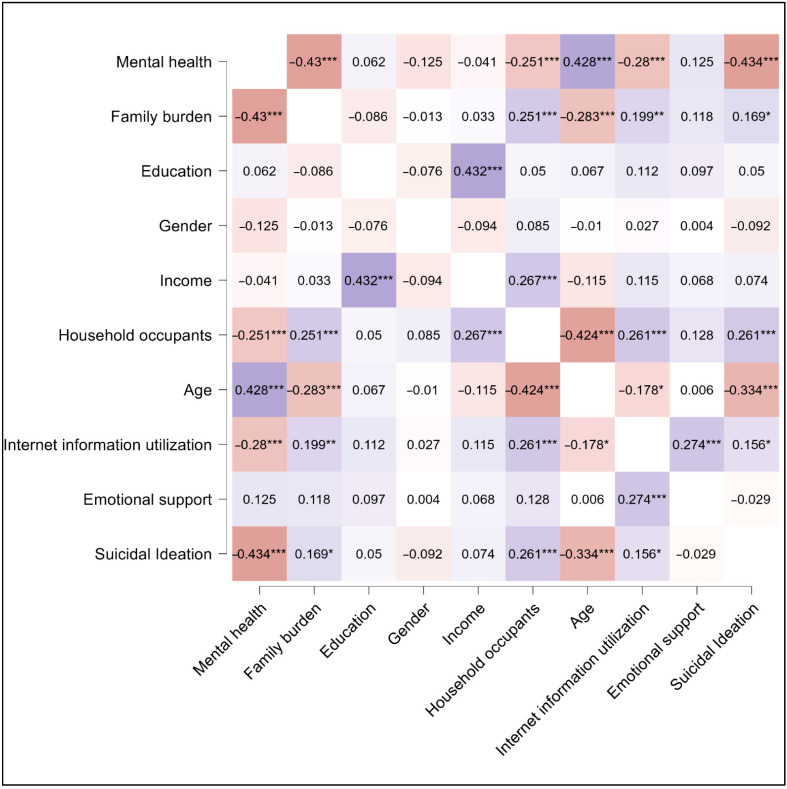
Correlation matrix of study variables. Shades of blue represent positive correlation and shades of brown represent negative correlation. The transparency of the color indicates the strength of the relationships; * *p* < 0.05; ** *p* < 0.01; *** *p* < 0.001.

In the examined cohort of cancer patients, our correlation analysis (Figure 6) delineated the relationship between several psychosocial factors and both mental health and suicidal ideation. A significant inverse correlation was found between mental health and suicidal ideation (r = −0.434, *p* < 0.001). Access to information and emotional support also played important roles. Internet information utilization was inversely related to suicidal ideation (r = −0.280), proposing that the ability to find health-related information online might serve as a protective factor against the development of suicidal thoughts. In contrast, emotional support was positively correlated with better mental health (r = 0.274), underscoring its importance in the psychosocial health of cancer patients.

The perceived burden on the family also appears to be a critical factor, as evidenced by its negative correlation with mental health (r = −0.430, *p* < 0.001), indicating that patients who feel their illness is a greater burden to their family may suffer more significant mental health challenges. This same perceived family burden showed a moderate positive correlation with suicidal ideation (r = 0.169), although this relationship was less pronounced. The number of household occupants was negatively correlated with mental health (r = −0.251), which may reflect the stress associated with larger household sizes, and this factor also showed a slight positive correlation with suicidal ideation (r = 0.118), suggesting a complex interaction between the patient’s immediate social environment and their mental well-being. Notably, age presented a robust negative correlation with suicidal ideation (r = −0.283), indicating that younger patients in this study were more prone to such thoughts compared to their older counterparts. This may reflect generational differences in dealing with illness or the accumulation of coping mechanisms over time.

### 3.3. Qualitative Findings from the Survey

In our qualitative exploration of the experiences of cancer patients, several interrelated themes emerged, each illuminated by the participants’ poignant narratives. These themes, as noted in Table 2, included (a) support, (b) care quality, (c) information, and (d) financial burdens. These findings offer a multifaceted perspective on the psychosocial challenges encountered by cancer patients and underscore the complexity of their mental health needs.

#### 3.3.1. Support

The theme of support covered a broad spectrum of experiences and sentiments shared by cancer patients and survivors. The feeling of connection and not being alone was important, as highlighted by Participant 68. The scary reality of feeling isolated during such a vulnerable time underscores the critical need for ongoing emotional and social support. This sentiment is echoed in the reflections of other participants, who underline the difference made by a supportive and caring medical staff. Participant 100, for example, notes the initial reassurance given by their physician, a fundamental cornerstone in building a hopeful and supportive treatment journey.

Participant 62’s statement on the duty of medical professionals to show genuine concern further emphasizes the necessity for healthcare providers not just to perform their clinical roles but to engage empathetically with patients. This engagement creates a supportive environment critical for patient well-being. Similarly, Participant 135’s call for the medical profession to actively engage in discussions about emotional support and to have support teams available for those in need points to a gap in the current care models. It suggests a move towards more holistic care approaches that recognize and address the emotional and psychological facets of battling cancer.

Participant 105’s experience offers a unique perspective on the personalization of care. The remark that medical professionals managed to make them feel like more than just another patient speaks volumes about the value of personable interactions in healthcare settings. It shows that professional distance does not preclude forming meaningful connections that can significantly enhance a patient’s experience.

The importance of post-treatment support is highlighted by Participant 189, who points out the necessity for continued outreach and support groups. This need reflects an understanding that the cancer journey does not simply end with the conclusion of medical treatment; the aftereffects can linger and require additional, often overlooked, forms of support.

Lastly, Participant 198’s reflection brings an essential aspect of patient autonomy and respect to the forefront. The desire for straightforward communication and not being coddled emphasizes the need for healthcare professionals to balance empathy with respect for the patient’s maturity and intelligence. Their regret at not engaging with available support groups also underscores the sometimes-overlooked value of these groups in providing mutual support and understanding among those undergoing similar challenges.

#### 3.3.2. Care Quality

The theme of care quality in the context of cancer treatment highlighted the critical nuances of personalized care and the impact it has on patients’ experiences and outcomes. Participant 65’s call to move away from a one-size-fits-all approach to treatment and recognize the uniqueness of each patient’s situation highlights a fundamental challenge within healthcare systems. This perspective underlines the importance of tailoring care to individual needs rather than adhering to overly standardized treatment protocols, which may not serve every patient effectively.

The distressing experience shared by Participant 2, where the lack of peace of mind and adequate treatment led to extreme despair, underscores the dire consequences of perceived inadequate care. This tragic account emphasizes the essential need for comprehensive, compassionate treatment strategies that address not only the physical aspects of cancer but also the psychological and emotional toll it exacts on individuals.

Participant 98’s experience with chemotherapy highlights the unpredictability of cancer treatments and the importance of responsive and adaptable care. The decision to halt treatment due to adverse reactions illustrates the necessity of closely monitoring patients’ responses and prioritizing their safety and well-being over rigid treatment regimens.

The suggestion from Participant 47 for more proactive referrals and resources from the onset of diagnosis points to a need for a more integrated care model. Early and comprehensive access to information, support, and additional resources can empower patients, help them navigate their treatment journey more effectively, and potentially improve outcomes.

Participant 2 also brings to light the benefits of holistic treatment approaches that consider the patient’s overall well-being. The contrast drawn between the adverse effects of conventional treatments like chemotherapy and radiation and the perceived efficacy and gentleness of holistic treatments speaks to a growing patient interest in integrative care models that blend medical and supportive therapies.

Participant 114’s experience reflects the significant role of nursing care in the treatment experience. The emphasis on continuous follow-up, quality care, and the personal touch provided by nurses showcases the critical impact of nursing staff in making complex treatment regimes more manageable and humanizing the healthcare experience.

#### 3.3.3. Information

The theme of information in cancer care emphasized the importance of transparent, comprehensive communication between healthcare providers and patients. This theme encompassed the need for clarity about treatment options, potential side effects, and the availability of support and resources. The experiences shared by the participants underscore a common desire for more detailed information that could better prepare them for the journey ahead.

Participant 46’s account of being uninformed about the potential side effects of radiation and chemotherapy treatments highlights a significant gap in pre-treatment counseling. This lack of upfront information deprived them of the opportunity to prepare mentally and physically for what was to come, illustrating the importance of setting realistic expectations from the outset.

Participant 175’s request for more resources and opportunities to connect with other survivors’ points to a need for comprehensive informational support that extends beyond the medical aspects of cancer treatment. Providing patients with access to survivor networks can offer invaluable peer support and insight, helping to alleviate fears and uncertainties.

The advice from Participant 19 to maintain a hopeful outlook while also being factual about treatment options and outcomes underscores the delicate balance required in communicating with cancer patients. Informing patients about support groups as part of this communication strategy further emphasizes the importance of integrating emotional and social support into the care pathway.

Participant 186’s suggestion for increased therapy use and clearer explanations from physicians about treatment processes and what to expect reflects a broader desire for a more involved and informative approach to patient care. This proactive involvement can help patients navigate their emotions, particularly during the anxiety-filled periods of waiting for test results.

Participant 192 calls for better explanations of both the positive aspects and potential side effects of treatments, highlighting the need for balanced information that addresses the benefits of treatment while also preparing patients for any adverse effects. Such balanced information can help manage expectations and reduce anxiety about the impact of treatment on quality of life.

Participant 197’s suggestion for openness about what to expect and for more accessible resources and contacts for shared experiences underscores a common theme: the desire for an open line of communication and a broader support network. This approach can help patients feel more informed and less isolated in their experiences.

Lastly, Participant 187’s call for education about the specifics of treatment and its potential success rates points to a fundamental need for clear, detailed information that empowers patients to make informed decisions about their care.

#### 3.3.4. Financial Burden

The theme of financial burden captured a distressing aspect of the cancer journey, highlighting the toll that treatment costs can impose on patients and their families. This theme surfaced concerns about the affordability of care, the impact of financial stress on mental well-being, and the desire for a healthcare system that offers more compassionate financial support.

Participant 107’s critique of the perceived coldness and lack of empathy from medical professionals regarding the financial aspects of cancer treatment underscores a significant disconnect. The emotional distress caused by facing high treatment costs can be exacerbated by an impersonal approach from healthcare providers. This account calls for a more empathetic acknowledgment of the financial strain patients endure, suggesting that compassion should extend to all facets of patient care, including financial counseling and support.

The reflection from Participant 179 on financial stress being a primary source of concern, overshadowing even the fear of mortality, brings to light the devastating impact of financial burdens. The dread of not being able to provide for one’s family or the prospect of leaving them in financial distress can deeply affect a patient’s motivation to pursue or continue treatment. This highlights the need for comprehensive financial support systems that address these fears and alleviate the added emotional burden.

Participant 131’s experience of lacking insurance at the time of diagnosis illustrates the stark realities many face in accessing and affording necessary care. The financial barriers to treatment can lead to existential despair, making the already daunting challenge of fighting cancer seem insurmountable. This narrative reinforces the urgent need for healthcare policies and systems that ensure no one is left to navigate these waters alone due to financial constraints.

Lastly, Participant 138’s account of the financial burden impacting both family and career emphasizes the far-reaching consequences of cancer treatment costs. While the support received for their medical and emotional needs was adequate, the financial implications lingered as a pervasive concern, affecting multiple areas of life. This story serves as a reminder of the importance of addressing the financial aspects of cancer care as an integral part of the overall treatment and support plan.

### 3.4. Case Study Findings—Patient Journey

The case studies explore the diverse cancer journeys of four patients, each facing unique diagnostic and treatment challenges. The first patient’s journey began with a routine colonoscopy leading to an unexpected lung cancer discovery, which was a secondary finding during the treatment process for metastatic colorectal cancer. The second patient experienced a series of complex medical events starting with prostate cancer, which, after several years, culminated in the discovery of squamous cell carcinoma and undisclosed colon cancer. The third patient received his diagnosis via a phone call after a colonoscopy, highlighting a lack of support beyond the immediate medical interventions. The fourth patient’s diagnosis of Hodgkin’s disease unexpectedly led to the discovery of a cancerous colon polyp during routine follow-ups.

This section organizes the patient journey under four categories: (a) initial diagnoses, (b) treatment, (c) emotions, and (d) communication and artificial intelligence. The narratives underscore the critical importance of comprehensive care, effective communication, and the need for ongoing surveillance even after primary treatment concludes. Each story uniquely illustrates the challenges of navigating healthcare systems, the emotional impacts of cancer diagnosis and treatment, and the potential benefits of integrating advanced technologies like AI to enhance patient care and coordination.

#### 3.4.1. Initial Diagnosis

Patient 1—The participant’s journey through cancer began unexpectedly during a routine colonoscopy, which itself was fraught with complications. “Kind of an odd way that they discovered that I had cancer,” he described the initial stages where preparation issues led to multiple attempts to clear his system. An incidental finding on an X-ray revealed abnormalities: “But she did say, there’s something in your X-ray in your lungs. There’s a couple of spots that just look kind of weird. You should probably have it checked out.” After the unusual findings in the X-ray, he was referred to a pulmonologist who, after further testing, delivered the bad news of metastatic colorectal cancer via a phone call. Surprisingly, the primary tumor was in his lungs with no evident disease in his colon. “But when she called me, it was certainly a surprise. I had just had a colonoscopy, and everything was clear. And I think I was kind of more stunned and just sort of shocked than anything else,” he recounted, emphasizing the unexpected nature of his diagnosis.

Patient 2: The participant’s medical journey began with a diagnosis of prostate cancer, which set the stage for a series of complex medical challenges. “I came down with prostate cancer in 2003. And during the treatment for prostate cancer, I got radiation […] went to the hospital. They filled me up with blood and set me on my way,” he described the immediate aftermath of his initial treatment, indicating the beginning of a long battle with cancer and related health issues. Thirteen years after his initial diagnosis, during a routine check-up, the participant’s condition took a sudden turn for the worse. “My cbc came back at seven. Got a call from my doctor. He said, get to the hospital immediately,” he recounted, highlighting the critical nature of his situation. This visit led to the discovery of a lump on his throat, previously dismissed as benign, which was revealed to be squamous cell carcinoma, head and neck, stage four, during an emergency room visit. The severity of his condition was underscored during an emergency room consultation, where a quick biopsy was performed. “It was squamous cell carcinoma, head and neck, stage four,” he learned, revealing the gravity of his illness. Despite this alarming diagnosis, his internal bleeding remained unaddressed, prompting him to seek further medical opinions. His trusted radiation oncologist, located far from his initial treatment center, helped uncover another overlooked issue: colon cancer. “He says, I got more bad news for you… You’ve got colon cancer,” indicating a significant oversight by his previous medical team.

Patient 3: The patient recounted receiving his cancer diagnosis via a phone call, a moment that marked the beginning of his journey into the complex world of cancer treatment. The diagnosis was unexpected, coming after a routine colonoscopy that initially seemed unremarkable. “Yeah, it was a phone call. I was not symptomatic or anything… and she just told me it was cancer,” he explained about the shock of discovering his condition through such an impersonal medium. The doctor who performed the colonoscopy delivered the news directly and facilitated the next steps. “She doesn’t want to lose any of her patients. So she said, ‘let’s get it done’,” he recounted, noting the doctor’s proactive approach in coordinating his immediate care, which led to surgery scheduled within a month of the diagnosis.

Patient 4: His experience began with a referral from his primary care physician suspecting Hodgkin’s disease, leading him to consult with a hematology oncologist. “My primary care physician at the time said, I think you may have Hodgkin’s disease, and if you do, I want you to see this,” the patient recounts. During a routine follow-up, a new and significant health issue was discovered by the patient’s gastroenterologist. “In the interim, my gastroenterologist found a polyp that was cancerous. And so I had the polyp removed and a chunk of my colon, fortunately, with what I had, it was contained and that was the end of that,” the patient recounted. This unexpected finding highlighted the importance of comprehensive care and regular check-ups beyond the primary disease focus.

#### 3.4.2. Treatment

Patient 1: The initial treatment approach involved surgical removal of the tumors, followed by rounds of chemotherapy and radiation during the first recurrence. However, as the cancer proved resistant, particularly to radiation, his treatment strategy shifted to managing the disease with ongoing cycles of chemotherapy. “So now radiation is off the table and they’ve kind of set me up for at this point, barring some breakthrough, I’m going to probably be just sort of an off and on cycle of chemotherapy,” he explained, depicting a regimen aimed at keeping the cancer at bay rather than achieving a cure.

Patient 2: Frustrated with the fragmented and insufficient care he received initially, the participant sought more personalized and effective treatment closer to home. The local doctors he consulted provided a stark contrast to his earlier experiences. “Not the original group of doctors… This other group of doctors, it’s just two doctors. They have taken the time. They have sat down. They explained to me what’s going on,” he praised the communicative and thorough approach of his new medical team, which planned a treatment that considered both his cancers.

Patient 3: During the waiting period, the patient met with a surgeon who provided details about the procedure. However, he received no additional support, such as mental health assistance or home support, which left him feeling unsupported in aspects beyond the physical treatment. “The meeting with the surgeon, he went over how the procedure went and what to expect, but that was the extent of it,” he described the brief and to-the-point interaction. Looking back, the patient recognized the importance of early preventative measures which he had neglected, attributing part of his situation to delaying recommended screenings. “What would have helped if I went and done the colonoscopy at age 50 like my general physician had asked, instead of waiting till I was 56,” he lamented, acknowledging his own role in the late diagnosis. The post-treatment phase involved minimal interaction with healthcare providers, apart from necessary follow-ups. His brief experience with chemotherapy was detrimental, leading to its discontinuation after just three days due to severe adverse effects. “Well, like I said, three days that I was actively on chemo… my system was shutting down,” he recalled the intense and brief encounter with chemotherapy.

Patient 4: The polyp was surgically removed, and fortunately, it was contained. This incident highlighted the importance of ongoing surveillance even after the primary cancer treatment concludes, showing how other health issues can surface or be discovered. The patient highlighted the essential coordination among his healthcare providers. “Fortunately for me, my gastroenterologist knew all the other docs… and he would always stop and see and talk to all of them,” he stated, showing the beneficial outcomes of effective communication among different specialists managing his health.

#### 3.4.3. Emotions

Patient 1: The emotional toll of dealing with recurrent cancer and continuous treatment significantly affected his mental health. Initially struck by shock and disbelief, he later experienced deepening depression as treatments progressed. “I didn’t really start to feel like the depression from it until I went into treatment. That’s where I think I kind of started to experience,” he shared, highlighting the psychological challenges accompanying physical treatments. Despite these difficulties, he found great comfort in the interactions with the nursing staff during chemotherapy, who provided much-needed support.

Patient 3: The patient expressed mixed feelings about the communication method of his diagnosis, pondering whether an in-person disclosure would have made a significant difference. Reflecting on the emotional impact, he shared, “For several days, it really didn’t seem real… It took a while to really sink in.” The uncertainty and intermittent hopeful news compounded his anxiety, particularly when post-surgery findings were not as positive as expected. Family played a crucial role in his coping strategy, as he relied heavily on his wife and children for support, rather than formal mental health resources. “It was mainly just talking to my wife and kids and stuff,” he said, highlighting a personal support system that was more significant than any professional mental health intervention offered.

Patient 4: The patient vividly describes the significant role communication played throughout his treatment journey. From the initial diagnosis, regular updates via email with his doctor, to discussions about the progress and adjustments in his treatment plan, effective communication was pivotal. “Fortunately, my doctor was very email responsive, so I kept in touch with him, told him what I was feeling,” he shares, illustrating the responsive and patient-centered approach of his healthcare provider. Support from family and the medical team was a recurring theme in his narrative. His recount of bringing food for the medical staff on treatment days paints a picture of creating a supportive community atmosphere. “We had brought food for the whole department… which became a tradition,” he mentioned, indicating how personal touches and support systems can positively impact the treatment experience.

#### 3.4.4. Communication and Artificial Intelligence

Patient 1: Reflecting on his experiences, the participant emphasized the need for improved communication and more patient-focused care from healthcare providers. He advocated for the integration of technology like AI to enhance patient care by making medical information more accessible and understandable. “It’s very interesting, I hear, obviously, AI is in the news these days… It’s interesting to see AI being used for something that might actually benefit society,” he noted, underscoring the potential of AI to help manage medical data and guide patients through clinical trials and treatment options.

Patient 2: Throughout his narrative, the participant emphasized the importance of informed patient advocacy and the potential role of AI in enhancing patient care. He sees AI as an essential tool for alleviating the fears, anxieties, and uncertainties of cancer patients by providing comprehensive information about treatment options, costs, and procedures. “Absolutely… That’s something that has to come to be, and it will allay a lot of the fears, anxiety, depression, concern of a cancer patient as to how do I pay for it, what is it, how’s it done, what am I going to go through?” His experience underscores the need for advancements in technology to support effective patient-centered care.

Patient 3: While the patient appreciated the medical interventions that potentially saved his life, he identified significant gaps in emotional and informational support. He suggested that advancements like AI could fill these gaps, offering accessible and reliable information and support, especially beneficial for those without a strong personal support network.

Patient 4: Reflecting on his experience, the patient underscores the importance of tailored communication and suggests improvements for future patient care. He advocates for healthcare providers to engage more deeply with patients about their preferences for receiving information and to ensure all treating doctors coordinate effectively. This narrative suggests a model of care that supports not only the clinical treatment of the disease but also the emotional and psychological well-being of the patient.

## 4. Discussion

### 4.1. Main Takeaway

Our research highlights several critical aspects of the mental health challenges faced by cancer patients, revealing that passive suicidal ideation is notably prevalent and significantly linked to increased risks of suicide attempts, underscoring the need for early intervention and dedicated support [35,36,37,38]. The study further highlights the post-treatment phase as unexpectedly distressing, often more so than the diagnosis itself, suggesting that current care models need to extend support well beyond the treatment period. Additionally, the complexities of family dynamics and household size play a significant role in patient mental health, with perceived burdens and larger household sizes correlating with poorer mental health outcomes. The use of internet and AI in managing health information presents a paradox, where the quality and presentation of information are crucial for patient mental health. Despite these insights being well recognized within the professional community, patients continue to encounter these challenges, indicating a gap between professional awareness and effective implementation of solutions. This gap calls for an integrated approach to care that not only addresses the medical but also the psychological and social needs of cancer patients, emphasizing the urgency for reforms in healthcare practices and policies to better support these individuals through their cancer journey.

### 4.2. Distressing Phases of Cancer Treatment and Recovery

The initial diagnosis of cancer often brings unexpected challenges and complexities that set the stage for a unique and deeply personal journey through treatment and recovery. For instance, Patient 1’s experience in our case study underscores the unpredictability of cancer detection, where the disease was identified incidentally during a routine procedure not originally intended to diagnose such a condition. Similarly, Patients 2 and 3 received their cancer diagnoses under sudden or critical circumstances, reflecting the intense and often shocking nature of encountering cancer. However, the end of active treatment does not signal an end to the challenges for cancer patients. Instead, the post-treatment period is frequently identified as the most distressing phase, surpassing even the initial shock of diagnosis in terms of mental and emotional strain. Such insights call for a reassessment of post-treatment care models to provide more comprehensive support during this critical phase. In 2022 Moura et al. also emphasized the need for early-stage post-cancer treatment care outside of regional cancer centers [39]. Therefore, our research advocates for a holistic and continuous model of care, integrating mental health as a fundamental aspect (not an optional extension) of cancer treatment and recovery, thereby ensuring comprehensive support for patients throughout their journey.

The finding that the post-treatment recovery period is frequently the most distressing phase, surpassing the initial diagnosis in terms of mental and emotional strain, is supported by Costanzo et al., who found that the adjustment to life after treatment for breast cancer can be distressing, despite the expectation of relief [40]. In 2019, a study by Trevino et al. highlighted the distress experienced by cancer survivors [41]. Additionally, in 2016, Borowski et al. discussed the influence of individual, family, and community-level factors on the mental health of survivors of colorectal cancer, indicating the multifaceted nature of distress in cancer survivors [42].

The journey from shock, then denial, to eventual acceptance is a common psychological pathway for many cancer patients. This adaptation process is often complex and multidimensional, necessitating structured psychological support, including pre-habilitation assessment before treatment, rehabilitation assessment post-treatment, and health promotion assessment at the end of treatment [43]. The narratives of our cohort reveal that there exists a notable gap in formal psychological care within the healthcare system. Despite the availability of evidence-based clinical practice guidelines for psychosocial care of cancer patients, there are challenges in the utilization of professional psychological care, indicating the need for improved implementation and accessibility of such services [44].

### 4.3. Internet Use, Artificial Intelligence, Mental Health, and Suicide Ideation

There is a nuanced relationship where internet information utilization correlates negatively with both mental health and suicidal ideation. At the same time, participants in our case study indicated an inclination towards the use of AI for cancer information. This trend underscores the importance of reliable, easily accessible medical information and AI-augmented tools to guide patients through the complexities of cancer care, enabling more personalized and informed decision making [45,46].

While seeking information online or via AI can be a coping mechanism that potentially diminishes suicidal ideation by reducing uncertainty and fear, it can simultaneously be linked to poorer mental health outcomes. This paradox could stem from various causes: individuals grappling with mental health challenges might turn to the internet for help, yet the information they find could exacerbate their anxiety, especially if it is complex, overwhelming, or grim. Contrary to expectations, the empowering effect of accessible information does not always translate into improved mental health. Instead, this correlation paints a picture where the quality and presentation of information are critical. Patients may suffer increased distress if the health information they encounter online is not well curated to their needs. This highlights the imperative for healthcare providers to guide patients toward information that is not only reliable and relevant but also presented in a way that is comprehensible and reassuring. By doing so, the internet’s vast resources can be harnessed to support mental health effectively, ensuring that patients benefit from online information without incurring additional psychological distress.

Our finding that the empowering effect of accessible information does not always translate into improved outcome (mental health) is supported by Diviani et al., who in 2015 found that low health literacy plays a role in the evaluation of online health information, indicating that individuals with lower health literacy may struggle to assess the quality of the information they encounter [47]. In 2021, Newman et al. suggested that the quality and accessibility of information are crucial for promoting mental health and well-being [48]. In the same line, Wong and Cheung in 2019 acknowledged the importance of understanding patients’ behaviors and needs along with the quality and presentation of information [49].

### 4.4. Social Support and Loneliness

The open-ended survey responses of GI cancer patients feature the critical role of social and emotional support in their cancer journey. The finding resonates with the existing literature in psycho-oncology, which emphasizes the protective role of social support against psychological distress in cancer patients [50,51]. Additionally, the majority of patients with gastrointestinal (GI) malignancies are older, highlighting the importance of understanding the unique support needs of this patient population [52]. Evidence also suggests that perceived social support may be more critical than actual support available or received in terms of quality of life outcomes [53]. Studies have also demonstrated that emotional support positively impacts mental health by decreasing a sense of loneliness in individual advanced GI cancer patients and caregivers [54]. The case study findings also shed light on the varied forms of support systems that cancer patients lean on. The narratives highlight the emotional support garnered from empathetic interactions with nurses, suggesting that these relationships play a crucial role in patient well-being [55,56]. Such empathetic approach is not only beneficial for emotional well-being but can also positively impact treatment outcomes [57]. It also illustrates the complexities of managing cancer within a family context and the challenges of self-managed mental health, underscoring the need for integrated care systems that include mental health services [57].

Our correlation analysis reveals significant insights into how cancer patients’ perceptions of their illness and the dynamics of their immediate social environment can impact their mental health and susceptibility to suicidal ideation. The negative correlation between the perceived burden on the family and mental health and suicide ideation are noteworthy, suggesting that cancer patients who feel that their illness imposes a significant burden on their family experience worse outcomes. This perception of being a burden can lead to feelings of guilt, helplessness, and worthlessness, exacerbating stress and potentially leading to depression and anxiety. These feelings not only impact the patient’s sense of self-worth but may also affect how they interact with their family members and other caregivers, potentially leading to a more isolated and less supportive care environment.

However, the observed negative correlation between the number of household occupants and mental health presents a contradiction to general intuition. Typically, more household members imply a more robust support network, potentially offering emotional and practical assistance that could alleviate some of the stress associated with illness. However, the contradiction observed in our analysis could have two possible explanations: the Simpson’s paradox or the financial burdens associated with maintaining a large family.

Simpson’s paradox occurs when a trend appears in several different groups of data but disappears or reverses when these groups are combined. In the context of household size and mental health, it is conceivable that larger households might seem beneficial in isolated analyses—such as within certain socio-economic brackets or specific family dynamics. However, when these groups are aggregated, the overall effect could be obscured or reversed due to underlying variables not accounted for in the initial analyses.

Managing a large family can introduce significant financial stress, especially if the family is dealing with the high costs associated with cancer treatment. The expenses related to healthcare, coupled with potential loss of income if the patient or primary caregivers are unable to work, can create a financial strain that outweighs the potential emotional benefits of having more household members. This stress could contribute negatively to the mental health of the patient, overshadowing the supportive aspects of a larger household. Addressing these factors in therapeutic settings and policy formulations could lead to more effective support systems that recognize the multifaceted nature of patient experiences within their family units.

### 4.5. Quality of Care

Patients’ perceptions of care quality and the desire for personalized treatment approaches highlight significant aspects of patient-centered care. The emphasis on individualized treatment resonates with the principles of person-centered therapy [58], suggesting that recognizing and addressing individual patient needs contributes significantly to treatment efficacy and patient satisfaction. The principles of person-centered therapy emphasize the importance of understanding and addressing individual patient needs [58]. In 2018, Loonen et al. discussed the significance of person-centered care in cancer survivorship, aiming to empower survivors and support self-management [59]. In 2020, Crabtree et al. reported the importance of recognizing individual needs in cancer survivorship [60]. Our case study findings also surfaced episodes of ineffective communication with healthcare providers, and the critical need for clear communication in disease management [61,62]. Holistic care, addressing both the physical and psychological needs of patients, emerges as a key element in improving the overall quality of cancer care. Thus, our study advocates for a balance between clinical efficiency and personalized care, as the de-personalization of treatment can negatively impact patient morale and trust in the healthcare system.

### 4.6. Transparency and Expenses

The significance of transparent and comprehensive information provision, as highlighted by the participants, reflects the concept of shared decision making and communication in healthcare. Patients’ desire for thorough information about treatment options, side effects, and outcomes points to the importance of informed consent and patient autonomy. The financial strain associated with cancer treatment also emerged as a profound stressor in our cohort. Existing studies have also associated financial strain with increased psychological symptoms in cancer patients, including anxiety and depression [63]. Additionally, the financial impact of cancer has been identified as a stressor that can precipitate posttraumatic stress disorder [64]. Patients’ concerns about financial burdens reflect the broader socio-economic context of healthcare, where studies have acknowledged relationships between lower socio-economic position and reduced chances of early stage cancer diagnosis, impacting treatment outcomes and survival [65].

Although previously documented in the literature and acknowledged by healthcare authorities and practitioners, these concerns persistently emerged in our cohort. The recurring nature of these issues suggests that they still need to be addressed in the current healthcare framework. Despite the awareness of these challenges within the medical community, there appears to be a gap between understanding these concerns and implementing practical solutions. This underscores an urgent need for further research and targeted intervention by medical experts and policymakers to enhance the patient experience and address these enduring concerns.

### 4.7. Recommendation

Healthcare providers should implement regular screenings for suicidal ideation as part of the oncological care routine. Additionally, training family in recognizing the subtle signs of passive ideation can enable earlier interventions. It is recommended that interventions involve family members to educate and reduce the stigma of being a burden. Support groups and family therapy can be effective in improving communication and reducing the stress associated with caregiving. Additionally, considering financial counseling and support for families dealing with the economic impacts of cancer care could alleviate some of the stress that contributes to poor mental health outcomes. Healthcare providers should recommend trusted websites and digital tools that provide accurate and relevant information, which can help in making informed decisions without overwhelming the patients. Moreover, training in digital literacy for both patients and caregivers can enhance the positive aspects of internet use while mitigating its potential drawbacks. To improve care quality and patient satisfaction, adopting person-centered approaches in treatment planning and communication is essential. This involves recognizing the unique needs of each patient and tailoring communication and care strategies accordingly. Transparent discussions about treatment costs should be part of the initial consultations and throughout the treatment process.

### 4.8. Limitations and Future Work

In addressing the limitations of our study, it is essential to consider several factors. The sampling method, primarily based on self-selection via a web-based survey distributed through social media and open cancer forums, may lead to selection bias and limit the generalizability of our findings to the broader population of individuals with GI cancer in the US. Furthermore, the reliance on self-reported data raises concerns regarding the accuracy and potential bias in participant responses, although a control question was included to enhance response integrity. The limited number of survey participants, and follow-up interviews, may not sufficiently represent the diverse experiences of the targeted population, thus potentially narrowing the scope of qualitative insights. The exclusive use of online platforms for data collection could inadvertently exclude individuals lacking internet access or digital literacy.

While this study provides critical insights into the psychosocial challenges faced by gastrointestinal (GI) cancer patients, several avenues remain for future exploration. First, longitudinal research is needed to monitor changes in mental health and suicidal ideation from diagnosis through treatment and survivorship. Such designs would allow for causal inferences and better understanding of when patients are most vulnerable, particularly during the post-treatment recovery phase identified as especially distressing in this study. Additionally, future studies should aim for larger and more diverse samples, including patients with varying cancer subtypes, stages, and treatment pathways, to improve generalizability and subgroup analyses.

Second, although this study highlights the protective roles of emotional support and internet information use, experimental or intervention-based studies are needed to evaluate how structured psychosocial interventions—such as digital mental health tools, AI-guided information systems, or family-focused counseling—can actively reduce distress and prevent suicidal ideation. Given the mixed impact of online information on mental health, future work should also explore how information quality, digital literacy, and trust in AI mediate these outcomes.

Third, expanding the qualitative component to include more in-depth interviews would help capture the nuanced experiences of underserved populations, such as racial and ethnic minorities or rural residents. Future work could also use mixed-method approaches integrating physiological data (e.g., sleep quality, cortisol levels) or ecological momentary assessments (EMA) to triangulate psychological distress with real-time behavioral indicators.

Finally, researchers and practitioners should collaborate to translate these findings into clinical practice through the development of screening protocols, post-treatment mental health plans, and training modules for oncology teams. This will help bridge the implementation gap between psychological awareness and action in cancer care.

## 5. Conclusions

Our study offers a comprehensive exploration of the psychosocial experiences of gastrointestinal cancer patients, providing valuable insights into their mental health challenges, coping mechanisms, and perceptions of healthcare communication and artificial intelligence in cancer care. Key findings revealed a notable prevalence of suicidal ideation, particularly in the more passive forms. However, the intensity of such thoughts decreased as they transitioned towards concrete planning, highlighting critical intervention points to prevent progression to more severe stages. The most significant mental distress was reported not at the time of diagnosis, as might be expected, but during the post-treatment recovery phase. This finding underscores the necessity for continuous psychological support extending beyond the completion of medical treatment.

The study also illuminated the critical role of psychosocial factors, such as family burden and emotional support, in influencing the mental health of GI cancer patients. Patients who perceived their illness as a substantial burden to their families experienced greater mental distress, emphasizing the importance of holistic care that includes family counseling and support services. Patients valued clear, empathetic communication and the provision of comprehensive, understandable information from their healthcare providers.

## Figures and Tables

**Table 1:** Descriptive statistics of study variables.

	Mean	Std. Error of Mean	95% Confidence Interval Mean		Std. Dev.	95% Confidence Interval Std. Dev.		Variance	95% Confidence Interval Variance		Skewness	Std. Error of Skewness	Min	Max
			Upper	Lower		Upper	Lower		Upper	Lower				
Suicidal Ideation														
SI 1	3.31	0.10	3.51	3.10	1.48	1.54	1.41	2.18	2.37	1.99	0.02	0.17	1	5
SI 2	3.64	0.11	3.85	3.43	1.54	1.60	1.45	2.36	2.56	2.10	−0.44	0.17	1	5
SI 3	3.94	0.11	4.15	3.73	1.50	1.60	1.38	2.25	2.57	1.90	−0.93	0.17	1	5
SI 4	3.92	0.11	4.14	3.71	1.55	1.66	1.42	2.40	2.75	2.01	−0.95	0.17	1	5
SI 5	3.92	0.11	4.13	3.70	1.52	1.61	1.41	2.32	2.60	1.99	−0.90	0.17	1	5
SI 6	3.89	0.11	4.10	3.68	1.54	1.64	1.43	2.38	2.67	2.03	−0.88	0.17	1	5
SI 7	4.03	0.11	4.24	3.83	1.49	1.61	1.34	2.23	2.60	1.81	−1.14	0.17	1	5
SI 8	4.08	0.10	4.28	3.87	1.48	1.59	1.33	2.18	2.54	1.76	−1.20	0.17	1	5
Mental Health														
MH 1	2.65	0.07	2.79	2.52	0.99	1.05	0.92	0.97	1.11	0.84	−0.20	0.17	1	4
MH 2	2.78	0.07	2.91	2.65	0.93	0.99	0.86	0.87	0.99	0.74	−0.19	0.17	1	4
MH 3	2.24	0.06	2.37	2.12	0.91	0.97	0.83	0.82	0.95	0.69	0.27	0.17	1	4
MH 4	2.76	0.07	2.90	2.62	1.00	1.06	0.93	1.01	1.13	0.86	−0.22	0.17	1	4
Household Occupants	2.51	0.09	2.70	2.33	1.33	1.49	1.15	1.77	2.22	1.33	1.24	0.17	1	8
Internet Information Utilization	3.11	0.09	3.29	2.93	1.31	1.38	1.23	1.70	1.91	1.51	0.04	0.17	1	5
Emotional Support	3.00	0.09	3.17	2.82	1.29	1.37	1.22	1.68	1.89	1.48	0.19	0.17	1	5
Family Burden	2.64	0.09	2.83	2.46	1.32	1.41	1.23	1.75	1.98	1.51	0.43	0.17	1	5

SI 1 = Have you thought about being dead or what it would be like to be dead? SI 2 = Have you wished you were dead or wished you could go to sleep and never wake up? SI 3 = Do you wish you were not alive anymore? SI 4 = Have you thought about doing something to make yourself not alive anymore? SI 5 = Have you had any thoughts about killing yourself? SI 6 = Have you thought about how you would make yourself not alive anymore (kill yourself)? SI 7 = When you thought about making yourself not alive anymore (or killing yourself), did you think that this was something you might do? SI 8 = Have you decided how or when you would make yourself not alive anymore/kill yourself? MH 1 = During your cancer treatment, did you ever experience loneliness? MH 2 = During your cancer treatment, did you ever experience hopelessness? MH 3 = During your cancer treatment, did you ever experience nervousness? MH 4 = During your cancer treatment, did you ever experience sever sadness? Internet information utilization = Have you ever used the internet to learn about your cancer and treatment plan. Emotional Support = During cancer treatment, did you ever share your thoughts and emotions with someone? Household Occupants = Including yourself, how many people live in your household?

**Table 2:** Sample quotes from the study for different participants.

Theme
Support
“Stay connected. The feeling of being alone. While I understand that most people don’t want to be a burden to the patient, the feeling of being alone can be a bit scary.” (participant ID 68)
“The first thing he told me was that we were going to cure it. All the staff were very supportive and caring. This is what a person needs.” (participant ID 100)
“Support during the course of treatment and actually doing their job, showing concern about their patients.” (participant ID 62)
“I think the medical profession needs to talk with patients to determine what emotional support they have and have support teams available for patients who aren’t as fortunate as I am.” (participant ID 135)
“Although professionals and this being their job not their actual life or way of such, it’s remarkable how personable they have been in not making me feel like just another patient.” (participant ID 105)
“Continue to reach out after care is done and make patient feel welcome to contact with aftereffects of treatment, especially radiation burns and pain. Make support group meeting available.” (participant ID 189)
“I wouldn’t want people to treat me like I am in kindergarten. I was an adult and just wanted the facts and a cure. There were available but I didn’t engage, and I really should have.” (participant ID 198)
Care quality
“Stop treating people like it’s a cattle call. One treatment doesn’t fit everyone. Show that every situation is unique and should be treated as such.” (participant ID 65)
“We needed better treatment, but there was no peace of mind, so we were forced to commit suicide.” (participant ID 2)
“My treatment was surgery followed by attempted Chemo. My body responded very negatively to the chemo and my doctor pulled me off after 3 days. From then on, I was only monitored but it did not come back.” (participant ID 98)
“There should be more referrals for resources from day 1 of the diagnosis or even during the testing phase.” (participant ID 47)
“To give holistic treatment it helps more than you know you don’t feel as bad as when the chemo or radiation treatment and it defiantly works better, and you don’t feel like crap either” (participant ID 2)
“Keep checking back with me and good quality care. Good nurses and limited visits to doctors. The nurses have made my treatment doable.” (participant ID 114)
Information
“There were a lot of side effects that my radiation and chemo caused that I wasn’t told about up front. Had I known this ahead of time I could have prepared myself for it.” (participant ID 46)
“Have more resources available for me to read about what to expect and a place I can go in person to talk to other survivors.” (participant ID 175)
“Be positive and upbeat there is always hope, give the information, tell the patient what the different treatments are, be factual, as to expected results, tell the patient about support groups.” (participant ID 19)
“Encourage more use of therapy and have physicians participate in treatment and clearly explain treatments(s) I had more emotions just before going back for follow up tests/scans and waiting for results.” (participant ID 186)
“Better explain the positive aspects of chemotherapy or any cancer treatment such as survival rate increases compared to no treatment at all. Also, better explain how side effects of any cancer treatment will be dealt with so the patient isn’t constantly worried about future side effects destroying their quality of life.” (participant ID 192)
“Be extremely open as to what I have and going to experience. Provide more information and resources. And who I could get in touch with who is experiencing what I’m dealing with.” (participant ID 197)
“Educating the patient on what exactly the treatment entails and how successful the treatment can be.” (participant ID 187)
Financial burden
“Medical professionals should be more caring to patients especially when they know that cancer treatment is so expensive. The attitudes that I ran into seemed to be cold and uncaring for their patients that were getting ready to see and be charged some of the highest bills in their life just to be kept alive.” (participant ID 107)
“Financial support is really the biggest stress especially in terms of my family. Thoughts of mortality and not seeing my children are the biggest emotional stress. You feel like why fight a losing battle.” (participant ID 179)
“I wish cancer wasn’t so money driven. I didn’t have insurance the first time I was diagnosed, and I stressed about how to stay alive. That’s when I first started feeling like life wasn’t worth living.” (participant ID 131)
“They did fine with caring and providing support as needed. The financial burden was great and effected my family and career.” (participant ID 138)

## Data Availability

The survey data presented in this study are available here: https://doi.org/10.5061/dryad.djh9w0wcf (Waiting for browsing permissions to be opened).

## Supplementary Materials

The following supporting information can be downloaded at: https://www.mdpi.com/article/10.3390/cancers17152460/s1, Reliability and validity outcomes.

## References

[1] Fincham B., Langer S., Scourfield J., Shiner M. (2012). Understanding Suicide: A Sociological Autopsy. Choice Rev. Online.

[2] Levi-Belz Y., Gvion Y., Apter A. (2019). The psychology of suicide: From research understandings to intervention and treatment. Front. Psychiatry.

[3] Sung H., Ferlay J., Siegel R.L., Laversanne M., Soerjomataram I., Jemal A., Bray F. (2021). Global cancer statistics 2020: GLOBOCAN estimates of incidence and mortality worldwide for 36 cancers in 185 countries. CA A Cancer J. Clin..

[4] Zalsman G., Hawton K., Wasserman D., van Heeringen K., Arensman E., Sarchiapone M., Carli V., Höschl C., Barzilay R., Balazs J. (2016). Suicide prevention strategies revisited: 10-year systematic review. Lancet Psychiatry.

[5] Bolton J.M., Gunnell D., Turecki G. (2015). Suicide risk assessment and intervention in people with mental illness. Bmj.

[6] Rawla P., Barsouk A. (2019). Epidemiology of gastric cancer: Global trends, risk factors and prevention. Prz. Gastroenterol..

[7] Bowden M.B., Walsh N.J., Jones A.J., Talukder A.M., Lawson A.G., Kruse E.J. (2017). Demographic and clinical factors associated with suicide in gastric cancer in the United States. J. Gastrointest. Oncol..

[8] Zaorsky N.G., Zhang Y., Tuanquin L., Bluethmann S.M., Park H.S., Chinchilli V.M. (2019). Suicide among cancer patients. Nat. Commun..

[9] Arnold M., Abnet C.C., Neale R.E., Vignat J., Giovannucci E.L., McGlynn K.A., Bray F. (2020). Global Burden of 5 Major Types of Gastrointestinal Cancer. Gastroenterology.

[10] Choudhury A. (2023). Impact of Social Isolation, Physician-Patient Communication, and Self-perception on the Mental Health of Patients With Cancer and Cancer Survivors: National Survey Analysis. Interact. J. Med. Res..

[11] Shahsavar Y., Choudhury A. (2023). Examining influential factors in newly diagnosed cancer patients and survivors: Emphasizing distress, self-care ability, peer support, health perception, daily life activity, and the role of time since diagnosis. PLoS ONE.

[12] Henson K.E., Brock R., Charnock J., Wickramasinghe B., Will O., Pitman A. (2019). Risk of Suicide After Cancer Diagnosis in England. JAMA Psychiatry.

[13] Kumar V., Chaudhary N., Soni P., Jha P. (2017). Suicide Rates in Cancer Patients in the Current Era in United States. Am. J. Psychiatry Resid. J..

[14] Holland J.C., Andersen B., Breitbart W.S., Buchmann L.O., Compas B., Deshields T.L., Dudley M.M., Fleishman S., Fulcher C.D., Greenberg D.B. (2013). Distress management. J. Natl. Compr. Cancer Netw..

[15] Nekhlyudov L., Mollica M.A., Jacobsen P.B., Mayer D.K., Shulman L.N., Geiger A.M. (2019). Developing a quality of cancer survivorship care framework: Implications for clinical care, research, and policy. JNCI J. Natl. Cancer Inst..

[16] Choudhury A., Shahsavar Y. (2023). Exploring the determinants influencing suicidal ideation and depression in gastrointestinal cancer patients. Sci. Rep..

[17] Lv G., Zhao D., Wang Q., Zhang M., Gao Y., Zhao X., Li P. (2022). Coping Style, Insomnia, and Psychological Distress Among Persons With Gastrointestinal Cancer. Nurs. Res..

[18] Yu T., Hu D., Jiang Y., Liu S. (2023). Influencing Factors of Suicidal Ideation in Lung Cancer Patients in Midland China: A Mixed-Method Study. Front. Psychiatry.

[19] Chiang Y.-C., Couper J., Chen J., Lin K.-J., Wu H.-P. (2021). Predictive Value of the Distress Thermometer Score for Risk of Suicide in Patients With Cancer. Support. Care Cancer.

[20] Posner K., Brent D., Lucas C., Gould M., Stanley B., Brown G., Fisher P., Zelazny J., Burke A., Oquendo M. (2008). Columbia-Suicide Severity Rating Scale (C-SSRS).

[21] Hesse B.W., Greenberg A.J., Peterson E.B., Chou W.S. (2017). The Health Information National Trends Survey (HINTS): A Resource for Consumer Engagement and Health Communication Research. Stud. Health Technol. Inform..

[22] Kroenke K., Spitzer R.L., Williams J.B., Löwe B. (2009). An ultra-brief screening scale for anxiety and depression: The PHQ-4. Psychosomatics.

[23] Bentler P.M. (1990). Comparative fit indexes in structural models. Psychol. Bull..

[24] Marsh H.W., Balla J.R., McDonald R.P. (1988). Goodness-of-fit indexes in confirmatory factor analysis: The effect of sample size. Psychol. Bull..

[25] Sivo S.A., Fan X., Witta E.L., Willse J.T. (2006). The search for” optimal” cutoff properties: Fit index criteria in structural equation modeling. J. Exp. Educ..

[26] Marsh H.W., Balla J.R., Hau K.-T., Marcoulides G.A., Schumacker R.E. (1996). An evaluation of incremental fit indices: A clarification of mathematical and empirical properties. Advanced Structural Equation Modeling Issues and Techniques, 1st ed.

[27] McDonald R.P., Marsh H.W. (1990). Choosing a multivariate model: Noncentrality and goodness of fit. Psychol. Bull..

[28] Cheung G.W., Wang C. (2017). Current Approaches for Assessing Convergent and Discriminant Validity with SEM: Issues and Solutions. Acad. Manag. Proc..

[29] Fornell C., Larcker D.F. (1981). Evaluating structural equation models with unobservable variables and measurement error. J. Mark. Res..

[30] Ab Hamid M., Sami W., Sidek M.M. Discriminant validity assessment: Use of Fornell & Larcker criterion versus HTMT criterion. Proceedings of the Journal of Physics: Conference Series.

[31] Carlberg C. (2014). Statistical Analysis: Microsoft Excel 2013.

[32] McAlister A.M., Lee D.M., Ehlert K.M., Kajfez R.L., Faber C.J., Kennedy M.S. Qualitative coding: An approach to assess inter-rater reliability. Proceedings of the 2017 ASEE Annual Conference & Exposition.

[33] Kendall J. (1999). Axial coding and the grounded theory controversy. West. J. Nurs. Res..

[34] Holton J.A. (2007). The coding process and its challenges. Sage Handb. Grounded Theory.

[35] Robson A., Scrutton F., Wilkinson L., Macleod F. (2010). The Risk of Suicide in Cancer Patients: A Review of the Literature. Psycho-Oncol..

[36] Liu R.T., Bettis A.H., Burke T.A. (2020). Characterizing the phenomenology of passive suicidal ideation: A systematic review and meta-analysis of its prevalence, psychiatric comorbidity, correlates, and comparisons with active suicidal ideation. Psychol. Med..

[37] Wastler H.M., Khazem L.R., Ammendola E., Baker J.C., Bauder C.R., Tabares J.V., Bryan A.O., Szeto E., Bryan C.J. (2022). An Empirical Investigation of the Distinction Between Passive and Active Ideation: Understanding the Latent Structure of Suicidal Thought Content. Suicide Life-Threat. Behav..

[38] May C.N., Overholser J.C., Ridley J., Raymond D. (2015). Passive Suicidal Ideation. Illn. Crisis Loss.

[39] Moura S., Nguyen P., Benea A., Townsley C. (2022). The Development and Implementation of the After Cancer Treatment Transition (ACTT) Program for Survivors of Cancer. Can. Oncol. Nurs. J..

[40] Costanzo E.S., Lutgendorf S.K., Mattes M.L., Trehan S., Robinson C., Tewfik F.A., Roman S. (2007). Adjusting to Life After Treatment: Distress and Quality of Life Following Treatment for Breast Cancer. Br. J. Cancer.

[41] Trevino K.M., Nelson C.J., Saracino R.M., Korc-Grodzicki B., Sarraf S., Shahrokni A. (2019). Is Screening for Psychosocial Risk Factors Associated With Mental Health Care in Older Adults With Cancer Undergoing Surgery?. Cancer.

[42] Borowski S., Siembida E.J., Nygren K., Bellizzi K.M. (2016). Correlates of Mental Health in Survivors of Colorectal Cancer: The Influence of Individual, Family, and Community Level Factors. J. Behav. Ther. Ment. Health.

[43] Shun S.C. (2016). Cancer Prehabilitation for Patients Starting From Active Treatment to Surveillance. Asia-Pac. J. Oncol. Nurs..

[44] Weis J., Hönig K., Bergelt C., Faller H., Brechtel A., Hornemann B., Stein B., Teufel M., Goerling U., Erim Y. (2018). Psychosocial Distress and Utilization of Professional Psychological Care in Cancer Patients: An Observational Study in N ational C omprehensive C ancer C enters (CCC s) in G ermany. Psycho-Oncol..

[45] McAllister M., Dunn G., Payne K., Davies L., Todd C. (2012). Patient Empowerment: The Need to Consider It as a Measurable Patient-Reported Outcome for Chronic Conditions. BMC Health Serv. Res..

[46] Akeel A.U., Mundy D. (2018). Re-Thinking Technology and Its Growing Role in Enabling Patient Empowerment. Health Inform. J..

[47] Diviani N., Putte B.v.d., Giani S., Weert J.C.M.v. (2015). Low Health Literacy and Evaluation of Online Health Information: A Systematic Review of the Literature. J. Med. Internet Res..

[48] Newman B., Fisher K.R., Trollor J.N. (2021). Right to Information for People With Intellectual Disability in Australian Mental Health Policy. J. Policy Pract. Intellect. Disabil..

[49] Wong D.K.-K., Cheung M.-K. (2019). Online Health Information Seeking and eHealth Literacy Among Patients Attending a Primary Care Clinic in Hong Kong: A Cross-Sectional Survey. J. Med. Internet Res..

[50] Zhang H., Zhao Q., Cao P., Ren G. (2017). Resilience and Quality of Life: Exploring the Mediator Role of Social Support in Patients With Breast Cancer. Med. Sci. Monit..

[51] Gu Z., Yang C., Tang L., Wu H. (2023). Interaction of Anxiety and Hypertension on Quality of Life Among Gynecological Cancer Patients: A Cross-Sectional Study. BMC Psychiatry.

[52] Rostoft S. (2017). Integration of Geriatric Assessment in the Care of Patients With Gastrointestinal Malignancies. Visc. Med..

[53] Applebaum A.J., Stein E., Lord-Bessen J., Pessin H., Rosenfeld B., Breitbart W. (2013). Optimism, Social Support, and Mental Health Outcomes in Patients With Advanced Cancer. Psycho-Oncol..

[54] Secinti E., Rand K.L., Johns S.A., O’Neil B.H., Helft P.R., Shahda S., Jalal S.I., Mosher C.E. (2018). Social Correlates of Mental Health in Gastrointestinal Cancer Patients and Their Family Caregivers: Exploring the Role of Loneliness. Support. Care Cancer.

[55] Liu R.N.J.-E., Mok R.N.E., Wong R.N.T. (2006). Caring in Nursing: Investigating the Meaning of Caring From the Perspective of Cancer Patients in Beijing, China. J. Clin. Nurs..

[56] Harding R.L., Beesley H., Holcombe C., Fisher J., Salmon P. (2015). Are Patient–nurse Relationships in Breast Cancer Linked to Adult Attachment Style?. J. Adv. Nurs..

[57] Zaker M.R., Safaripour A., Sabegh S.R.Z., Barjasteh S. (2022). Supportive Intervention Challenges for Patients With Breast Cancer: A Systematic Review. Asian Pac. J. Environ. Cancer.

[58] Hui K.-K., Hui E.K., Johnston M.F. (2006). The potential of a person-centered approach in caring for patients with cancer: A perspective from the UCLA center for East-West medicine. Integr. Cancer Ther..

[59] Loonen J.J., Blijlevens N.M.A., Prins J.B., Dona D., Hartogh J.d., Senden T., Broeder E.v.D.d., Velden K.v.d., Hermens R. (2018). Cancer Survivorship Care: Person Centered Care in a Multidisciplinary Shared Care Model. Int. J. Integr. Care.

[60] Crabtree B.F., Miller W.L., Howard J., Rubinstein E.B., Tsui J., Hudson S.V., O’Malley D., Ferrante J.M., Stange K.C. (2020). Cancer Survivorship Care Roles for Primary Care Physicians. Ann. Fam. Med..

[61] Torres-Blasco N., Costas-Muñíz R., Zamore C., Porter L.S., Claros M., Bernal G., Shen M.J., Breitbart W., Rosario L., Peña-Vargas C. (2022). Family as a Bridge to Improve Meaning in Latinx Individuals Coping With Cancer. Palliat. Med. Rep..

[62] Chan E.A., Tsang P.L., Ching S.S.Y., Wong F.Y., Lam W. (2019). Nurses’ Perspectives on Their Communication With Patients in Busy Oncology Wards: A Qualitative Study. PLoS ONE.

[63] Massicotte V., Ivers H., Savard J. (2021). COVID-19 Pandemic Stressors and Psychological Symptoms in Breast Cancer Patients. Curr. Oncol..

[64] French-Rosas L., Moye J., Naik A.D. (2011). Improving the Recognition and Treatment of Cancer-Related Posttraumatic Stress Disorder. J. Psychiatr. Pract..

[65] Rosskamp M., Verbeeck J., Gadeyne S., Verdoodt F., Schutter H.D. (2021). Socio-Economic Position, Cancer Incidence and Stage at Diagnosis: A Nationwide Cohort Study in Belgium. Cancers.

