# Supplementary material for: Exploring Patient-Centered Perspectives on Suicidal Ideation: A Mixed-Methods Investigation in Gastrointestinal Cancer Care
Source: Cancers (Basel). 2025 Jul 25;17(15):2460. doi: 10.3390/cancers17152460 (PMC12346693; doi:10.3390/cancers17152460)
Supplement: Supplementary file 1 [file cancers-17-02460-s001.zip › cancers-3726915-supplementary.pdf]

Results

Confirmatory Factor Analysis

Model fit

Chi-square test

Model	X^2	df	p
Baseline model	2176.35	132	
Factor model	297.89	104	< .001

Note. The estimator is ML.

Additional fit measures

Fit indices

Index	Value
Comparative Fit Index (CFI)	0.91
Tucker-Lewis Index (TLI)	0.88
Bentler-Bonett Non-normed Fit Index (NNFI)	0.88
Bentler-Bonett Normed Fit Index (NFI)	0.86
Parsimony Normed Fit Index (PNFI)	0.68
Bollen's Relative Fit Index (RFI)	0.83
Bollen's Incremental Fit Index (IFI)	0.91
Relative Noncentrality Index (RNI)	0.91

Information criteria

	Value
Log-likelihood	−3121.52
Number of free parameters	76.00
Akaike (AIC)	6395.04
Bayesian (BIC)	6646.47
Sample-size adjusted Bayesian (SSABIC)	6405.68

Other fit measures

Metric	Value
Root mean square error of approximation (RMSEA)	0.14
RMSEA 90% CI lower bound	0.12
RMSEA 90% CI upper bound	0.15
RMSEA p-value	3.98×10^{-13}
Standardized root mean square residual (SRMR)	0.05
Hoelter's critical N (α = .05)	88.34
Hoelter's critical N (α = .01)	96.25
Goodness of fit index (GFI)	0.95
McDonald fit index (MFI)	0.62
Expected cross validation index (ECVI)	2.23

Kaiser-Meyer-Olkin (KMO) test

	MSA	
Indicator	Yes	No
TT	0.76	0.91
UU	0.74	0.81
VV	0.93	0.88
WW	0.85	0.80
W	0.86	0.93
X	0.90	0.93
Y	0.88	0.90
Z	0.87	0.86
AA	0.87	0.92
BB	0.92	0.80
CC	0.87	0.81
DD	0.86	0.78
Overall	0.86	0.85

Bartlett's test of sphericity

Yes			No		
X^2	df	p	X^2	df	p
912.52	66	< .001	1132.97	66	< .001

R-Squared

	R^2	
	Yes	No
TT	0.69	0.39
UU	0.90	0.82
VV	0.53	0.30
WW	0.74	0.75
W	0.26	0.22
X	0.51	0.55
Y	0.66	0.80
Z	0.83	0.81
AA	0.89	0.78
BB	0.85	0.77
CC	0.88	0.65
DD	0.85	0.57

Parameter estimates

Factor loadings

Group	Factor	Indicator	Estimate	Std. Error	z-value	p	95% Confidence Interval		Std. Est. (all)
							Lower	Upper	
Yes	Mental Health	TT	0.85				0.69	0.98	0.83
		UU	0.87				0.74	0.99	0.95
		VV	0.66				0.50	0.80	0.73
		WW	0.93				0.78	1.05	0.86
	Factor 2	W	0.75				0.49	0.98	0.51
		X	1.12				0.83	1.32	0.71
		Y	1.23				0.89	1.47	0.81
		Z	1.43				1.13	1.62	0.91
		AA	1.42				1.13	1.62	0.94
		BB	1.41				1.17	1.61	0.92
		CC	1.38				1.10	1.61	0.94
		DD	1.38				1.04	1.61	0.92
No	Mental Health	TT	0.60				0.45	0.74	0.62
		UU	0.84				0.73	0.95	0.90
		VV	0.49				0.31	0.65	0.55
		WW	0.82				0.70	0.92	0.87
	Factor 2	W	0.68				0.45	0.88	0.47
		X	1.12				0.91	1.28	0.74
		Y	1.33				1.15	1.47	0.89
		Z	1.38				1.19	1.53	0.90
		AA	1.34				1.14	1.49	0.88
		BB	1.34				1.13	1.51	0.87
		CC	1.21				0.94	1.43	0.81
		DD	1.10				0.83	1.33	0.76

Factor variances

Group	Factor	Estimate	Std. Error	z-value	p	95% Confidence Interval		Std. Est. (all)
						Lower	Upper	
Yes	Mental Health	1.00	0.00			1.00	1.00	1.00
	Factor 2	1.00	0.00			1.00	1.00	1.00
No	Mental Health	1.00	0.00			1.00	1.00	1.00
	Factor 2	1.00	0.00			1.00	1.00	1.00

Factor Covariances

Group				Estimate	Std. Error	z-value	p	95% Confidence Interval		Std. Est. (all)
								Lower	Upper	
Yes	Mental Health	↔	Factor 2	0.43				0.23	0.60	0.43
No	Mental Health	↔	Factor 2	0.50				0.33	0.65	0.50

Residual variances

Group	Indicator	Estimate	Std. Error	z-value	p	95% Confidence Interval Lower	Upper	Std. Est. (all)
Yes	TT	0.32				0.18	0.50	0.31
	UU	0.08				0.01	0.15	0.10
	VV	0.39				0.23	0.55	0.47
	WW	0.30				0.17	0.41	0.26
	W	1.65				1.24	1.99	0.74
	X	1.21				0.64	1.83	0.49
	Y	0.79				0.29	1.38	0.34
	Z	0.40				0.07	0.91	0.17
	AA	0.25				0.01	0.71	0.11
	BB	0.37				0.02	0.88	0.15
	CC	0.26				0.05	0.50	0.12
	DD	0.34				0.04	0.71	0.15
No	TT	0.57				0.40	0.72	0.61
	UU	0.16				0.05	0.27	0.18
	VV	0.56				0.41	0.72	0.70
	WW	0.22				0.10	0.35	0.25
	W	1.67				1.38	1.93	0.78
	X	1.04				0.66	1.41	0.45
	Y	0.44				0.21	0.68	0.20
	Z	0.44				0.18	0.76	0.19
	AA	0.52				0.20	0.89	0.22
	BB	0.55				0.24	0.90	0.23
	CC	0.78				0.37	1.23	0.35
	DD	0.92				0.54	1.32	0.43

Factor Intercepts

Group	Factor	Estimate	Std. Error	z-value	p	95% Confidence Interval Lower	Upper	Std. Est. (all)
Yes	Mental Health	0.00	0.00			0.00	0.00	0.00
	Factor 2	0.00	0.00			0.00	0.00	0.00
No	Mental Health	0.00				−0.04	0.04	0.00
	Factor 2	0.00				−0.08	0.08	0.00

Intercepts

						95% Confidence Interval		
Group	Indicator	Estimate	Std. Error	z-value	p	Lower	Upper	Std. Est. (all)
Yes	TT	2.64				2.42	2.87	2.59
	UU	2.70				2.49	2.91	2.94
	VV	2.17				1.97	2.38	2.39
	WW	2.61				2.37	2.84	2.42
	W	3.41				3.08	3.75	2.29
	X	3.67				3.32	4.01	2.34
	Y	3.97				3.63	4.30	2.63
	Z	3.99				3.63	4.33	2.56
	AA	4.04				3.70	4.37	2.68
	BB	3.97				3.62	4.30	2.58
	CC	4.08				3.74	4.39	2.76
	DD	4.08				3.74	4.39	2.73
No	TT	2.66				2.50	2.81	2.77
	UU	2.83				2.70	2.97	3.04
	VV	2.29				2.14	2.43	2.54
	WW	2.86				2.72	2.99	3.04
	W	3.25				3.01	3.48	2.22
	X	3.62				3.41	3.82	2.39
	Y	3.92				3.75	4.09	2.64
	Z	3.88				3.71	4.06	2.53
	AA	3.84				3.66	4.02	2.53
	BB	3.84				3.66	4.03	2.50
	CC	4.01				3.82	4.20	2.67
	DD	4.08				3.89	4.28	2.79

Implied covariance matrices

Yes

TT	UU	VV	WW	W	X	Y	Z	AA	BB	CC	DD
1.04											
0.74	0.84										
0.56	0.58	0.83									
0.79	0.81	0.62	1.16								
0.27	0.28	0.21	0.30	2.22							
0.40	0.41	0.31	0.44	0.84	2.46						
0.44	0.46	0.35	0.48	0.92	1.37	2.29					
0.51	0.53	0.40	0.56	1.07	1.59	1.75	2.43				
0.51	0.53	0.40	0.56	1.07	1.59	1.74	2.03	2.27			
0.51	0.53	0.40	0.56	1.06	1.58	1.73	2.02	2.01	2.37		
0.50	0.51	0.39	0.55	1.04	1.54	1.70	1.97	1.97	1.96	2.18	
0.50	0.51	0.39	0.54	1.04	1.53	1.69	1.96	1.96	1.95	1.91	2.23

No

TT	UU	VV	WW	W	X	Y	Z	AA	BB	CC	DD
0.92											
0.50	0.87										
0.29	0.42	0.81									
0.49	0.69	0.40	0.88								
0.21	0.29	0.17	0.28	2.14							
0.34	0.48	0.28	0.46	0.76	2.28						
0.40	0.56	0.33	0.55	0.90	1.48	2.20					
0.42	0.59	0.34	0.57	0.94	1.55	1.83	2.36				
0.40	0.57	0.33	0.55	0.91	1.49	1.77	1.85	2.31			
0.40	0.57	0.33	0.55	0.92	1.50	1.78	1.86	1.80	2.36		
0.36	0.52	0.30	0.50	0.83	1.35	1.60	1.68	1.62	1.63	2.25	
0.33	0.47	0.27	0.45	0.75	1.23	1.46	1.53	1.48	1.48	1.34	2.14

Average variance extracted

Group	Factor	AVE
Yes	Mental Health	0.72
	Factor 2	0.71
No	Mental Health	0.57
	Factor 2	0.65

Heterotrait-monotrait ratio

Group		Mental Health	Factor 2
Yes	Mental Health	1.00	
	Factor 2	0.48	1.00
No	Mental Health	1.00	
	Factor 2	0.51	1.00

Reliability

Group		Coefficient ω	Coefficient α
Yes	Mental Health	0.91	0.91
	Factor 2	0.94	0.95
	total	0.92	0.93
No	Mental Health	0.82	0.83
	Factor 2	0.93	0.93
	total	0.93	0.91

Residual covariance matrices

Yes

TT	UU	VV	WW	W	X	Y	Z	AA	BB	CC	DD
< .001											
6.48×10^{-3}	< .001										
0.04	< .001	< .001									
< .001	5.71×10^{-3}	0.02	< .001								
0.19	0.20	0.26	0.40	< .001							
0.14	0.13	0.12	0.29	0.53	< .001						
8.56×10^{-3}	< .001	0.04	0.03	0.29	0.26	< .001					
0.14	< .001	0.02	0.10	0.01	< .001	< .001	< .001				
0.03	< .001	0.01	0.07	< .001	0.09	< .001	0.09	< .001			
0.09	< .001	0.08	0.06	< .001	< .001	< .001	0.04	< .001	< .001		
< .001	< .001	< .001	0.06	< .001	< .001	< .001	< .001	< .001	0.06	< .001	
< .001	< .001	< .001	< .001	< .001	< .001	0.22	< .001	< .001	< .001	0.04	< .001

No

TT	UU	VV	WW	W	X	Y	Z	AA	BB	CC	DD
< .001											
< .001	< .001										
0.07	< .001	< .001									
< .001	3.11×10^{-3}	0.01	< .001								
0.22	0.04	0.04	0.04	< .001							
0.19	0.05	0.08	0.10	0.16	< .001						
0.02	0.07	< .001	< .001	0.02	0.12	< .001					
0.03	0.03	< .001	< .001	< .001	< .001	0.05	< .001				
< .001	< .001	< .001	< .001	0.08	< .001	< .001	1.76×10^{-3}	< .001			
< .001	< .001	< .001	< .001	< .001	< .001	< .001	0.08	0.15	< .001		
0.11	0.14	0.12	3.09×10^{-3}	< .001	< .001	0.13	< .001	< .001	< .001	< .001	
< .001	< .001	< .001	< .001	< .001	< .001	< .001	< .001	< .001	0.13	0.41	< .001

Plots

Model plots

Yes

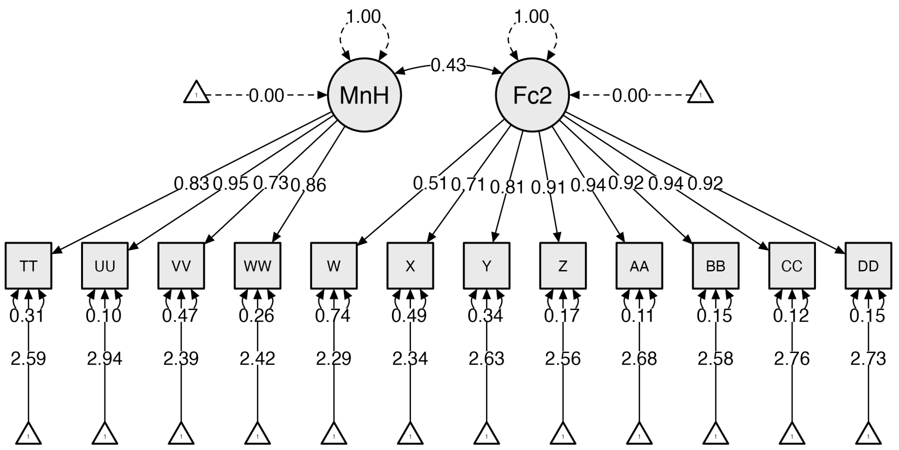

No

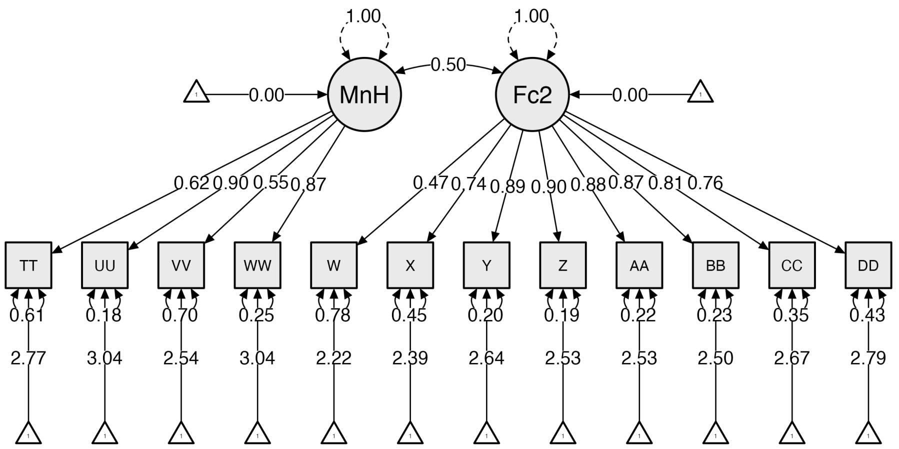

Misfit plots

Yes

	TT	UU	VV	WW	W	X	Y	Z	AA	BB	CC	DD
TT	0											
UU	.01	0										
VV	.04	.02	0									
WW	.04	.01	.02	0								
W	.12	.15	.19	.25	0							
X	.09	.09	.09	.17	.23	0						
Y	.01	0	.03	.02	.13	.11	0					
Z	.09	.01	.01	.06	.01	.01	.08	0				
AA	.02	.01	.01	.05	.06	.04	.02	.04	0			
BB	.05	.01	.06	.04	0	.06	.02	.01	.01	0		
CC	.05	.02	.03	.04	.02	.03	.02	0	.02	.02	0	
DD	.05	.09	.05	.01	.02	.02	.1	.04	0	.01	.02	0

No

	TT	UU	VV	WW	W	X	Y	Z	AA	BB	CC	DD
TT	0											
UU	.01	0										
VV	.09	.02	0									
WW	0	0	.01	0								
W	.16	.03	.03	.03	0							
X	.13	.03	.06	.07	.07	0						
Y	.01	.05	.06	.02	.01	.05	0					
Z	.02	.02	.04	.03	.04	.01	.02	0				
AA	.01	.01	.09	.08	.04	.02	.02	0	0			
BB	.02	0	.08	.06	.02	.02	.06	.03	.07	0		
CC	.07	.1	.09	0	.04	.02	.06	.02	.04	.08	0	
DD	.01	.01	0	.09	.01	.02	.02	.09	.02	.06	.19	0
